# A case report of duodenal arteriovenous malformation: usefulness of intraoperative indocyanine green angiography for precise identification of the lesion

**DOI:** 10.1186/s40792-021-01356-8

**Published:** 2022-01-04

**Authors:** Yoshihiro Kurata, Koichi Hayano, Keisuke Matsusaka, Hisashi Mamiya, Masaya Uesato, Kentaro Murakami, Masayuki Kano, Takeshi Toyozumi, Yasunori Matsumoto, Hiroshi Suito, Tetsuro Isozaki, Gaku Ohira, Hideki Hayashi, Hisahiro Matsubara

**Affiliations:** 1 Department of Frontier Surgery, Graduate School of Medicine, Chiba University, 1-8-1 Inohana, Chuo-ku, Chiba City, 260-8677 Japan; 2 Department of Molecular Oncology, Graduate School of Medicine, Chiba University, Chiba City, Japan; 3 Center for Frontier Medical Engineering, Chiba University, Chiba City, Japan

**Keywords:** Duodenum, Arteriovenous malformation, Indocyanine green

## Abstract

**Background:**

Arteriovenous malformation (AVM) of the gastrointestinal (GI) tract can cause bleeding. The treatment choice for GI tract AVM is surgical resection of the involved bowel segment with complete resection of the nidus. The AVM formed in the duodenum or pancreatic head could also cause gastrointestinal bleeding, and there are several reports of pancreaticoduodenectomy as its treatment. However, if the area of AVM can be accurately identified during surgery, it may be possible to completely resect the AVM while preserving the organ. We report a case of duodenal AVM in a patient successfully treated with a subtotal stomach-preserving duodenal bulb resection using intraoperative indocyanine green (ICG) angiography technique.

**Case presentation:**

An 18-year-old man was diagnosed with duodenal AVM after several examinations for anemia and was referred to our hospital for further treatment. Preoperative imaging studies showed that the inflow vessels of this duodenal AVM were the inferior pyloric artery and the superior duodenal artery, and the AVM was localized to the duodenal bulb. Thereafter, stomach-preserving duodenal bulb resection preceded by ligation of the inflow vessels was performed. During the surgery, ICG angiography clearly demonstrated the area, where the nidus was distributed, and a duodenal bulb resection with complete resection of the AVM was successfully performed. There was no recurrence at the 6-month follow-up.

**Conclusions:**

Intraoperative ICG angiography was a useful procedure for precise identification of the AVM of the GI tract.

## Background

Gastrointestinal (GI) arteriovenous malformation (AVM) sometimes causes GI bleeding [1, 2], and the radical treatment for AVM is complete surgical removal of the nidus lesion. It is important to accurately identify the lesion and resect the appropriate area, because resection with sufficient margins is overly invasive, and resection with insufficient margins leads to disease recurrence. Herein, we report a duodenal AVM case in which we determined the optimal surgical margins using intraoperative indocyanine green (ICG) angiography.

## Case presentation

**Fig. 1:**
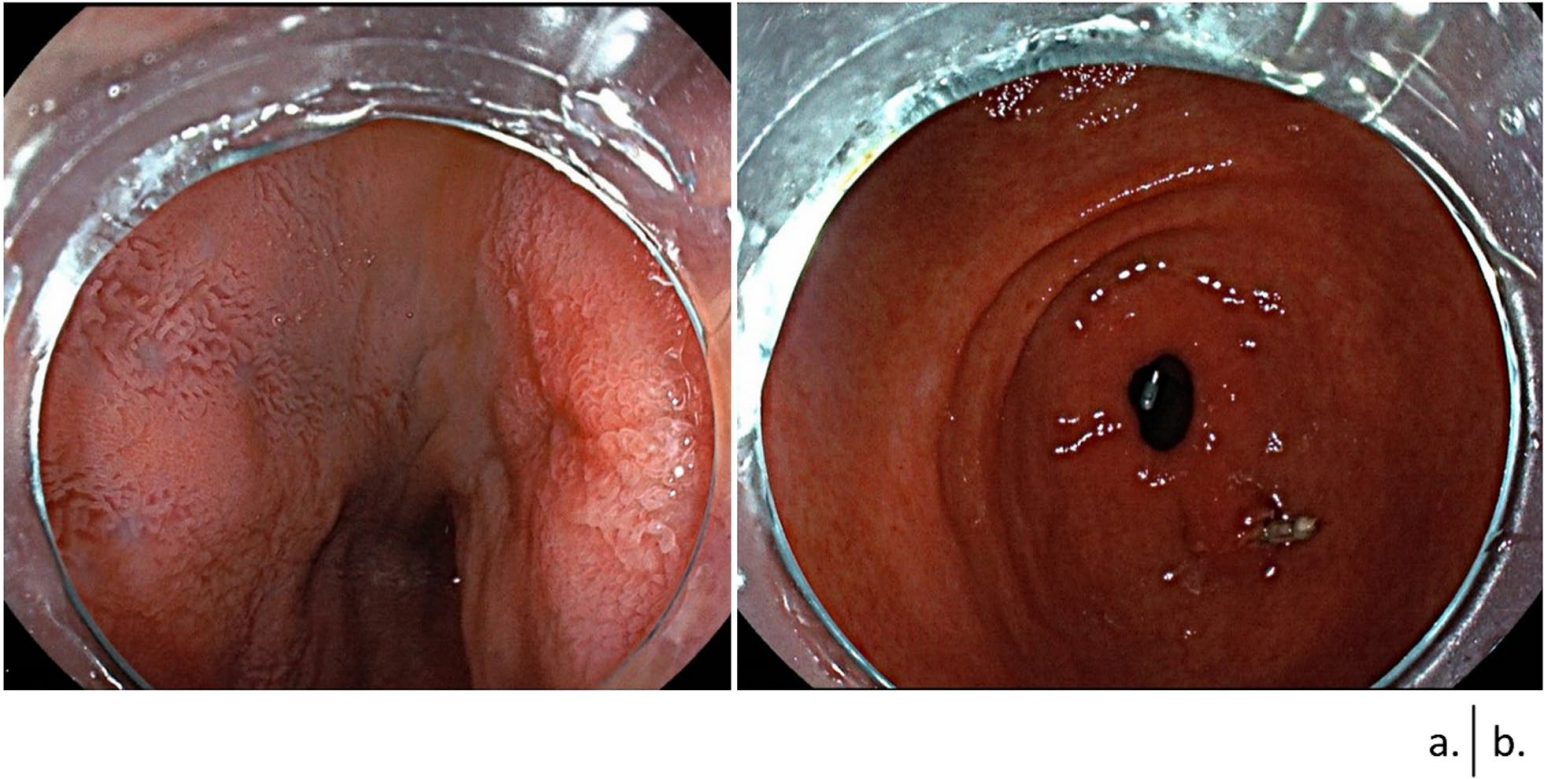
Preoperative endoscopic examination findings. **a** Upper gastrointestinal endoscopy findings showing erosions of the duodenal mucosa and pale veins running under the mucosa at the duodenal bulb. **b** No abnormalities are observed in the stomach

**Fig. 2:**
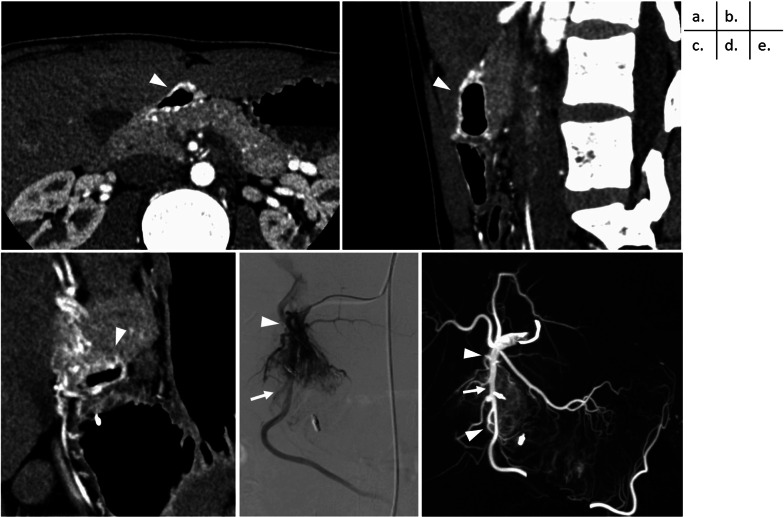
Preoperative contrast-enhanced computed tomography (CECT) and angio-CT images. **a**–**c** CECT images showing a hyper-enhancing duodenal bulb wall (arrowhead). No nidus is found in the pancreatic head. (**a** cross section, **b** sagittal section, **c** coronal section). **d** Angiography shows the arteries flowing into the duodenal AVM [arrow: inferior pyloric artery (IPA), arrowhead: superior duodenal artery (SDA)]. **e** Angio-CT images also show that the inferior pyloric artery (IPA) and superior duodenal artery (SDA) branching off from the gastroduodenal artery (GDA) are the inflow vessels. (arrow: GDA; arrowhead: IPA and SDA)

An 18-year-old man visited the previous hospital with a chief complaint of black stool and lightheadedness. He had a past medical history of surgery for a congenital skin tag on his buttock when he was 5 years and was noted to have congenital dysplasia of the sacrum. When he visited the hospital, severe anemia (hemoglobin level, 5.6 g/dL) was identified. Upper and lower endoscopy revealed erosions of the duodenal mucosa, and capsule endoscopy revealed a small amount of bleeding in the duodenum bulb. Finally, the angiography revealed duodenal AVM. There were no other lesions in the GI tract that could cause bleeding or anemia, and it was considered that a small amount of continuous bleeding from the duodenal AVM was the cause of the anemia. Thereafter, he was transferred to our hospital for further examination and treatment. Upper GI endoscopy revealed mucosal color changes in the duodenal bulb (Fig. 1). Contrast-enhanced computed tomography (CT) revealed a hyper-enhancing duodenal bulb wall, but no other enhancing lesions suspected as AVMs were detected in other areas of the body. Angio-CT findings suggested that the inflow vessel was the inferior pyloric artery (IPA) and superior duodenal artery (SDA), which branches off from the gastroduodenal artery (Fig. 2).

**Fig. 3:**
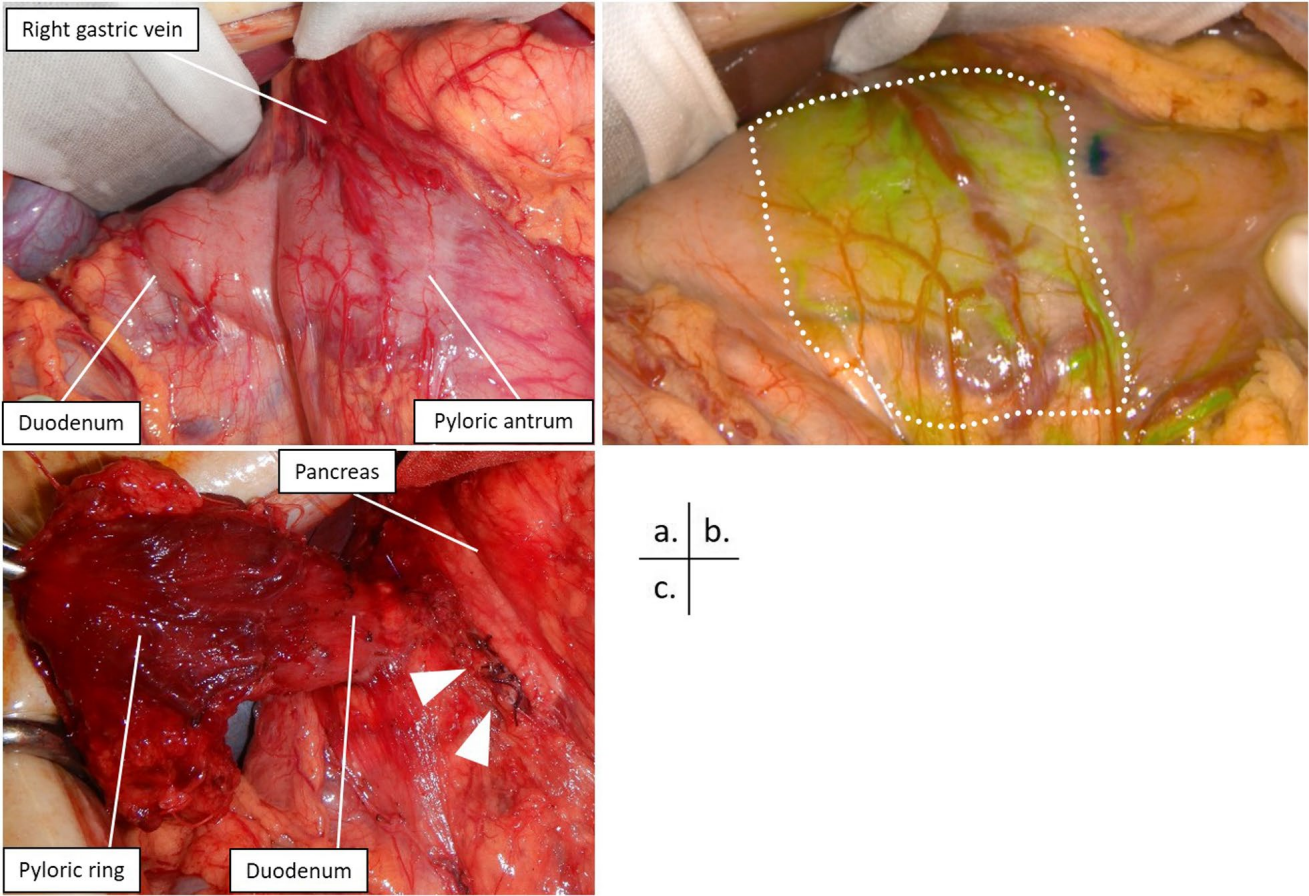
Intraoperative images. **a** Dilatation of the right gastric vein is found. Duodenal appearance has no remarkable change. **b** Indocyanine green is injected and observed with the infrared camera. The lesion and the boundary of the arteriovenous malformation are made clear (dotted line area). **c** Inflow vessels branching from the gastroduodenal artery are ligated (arrowheads), and the area from the pyloric ring to the duodenum is resected

**Fig. 4:**
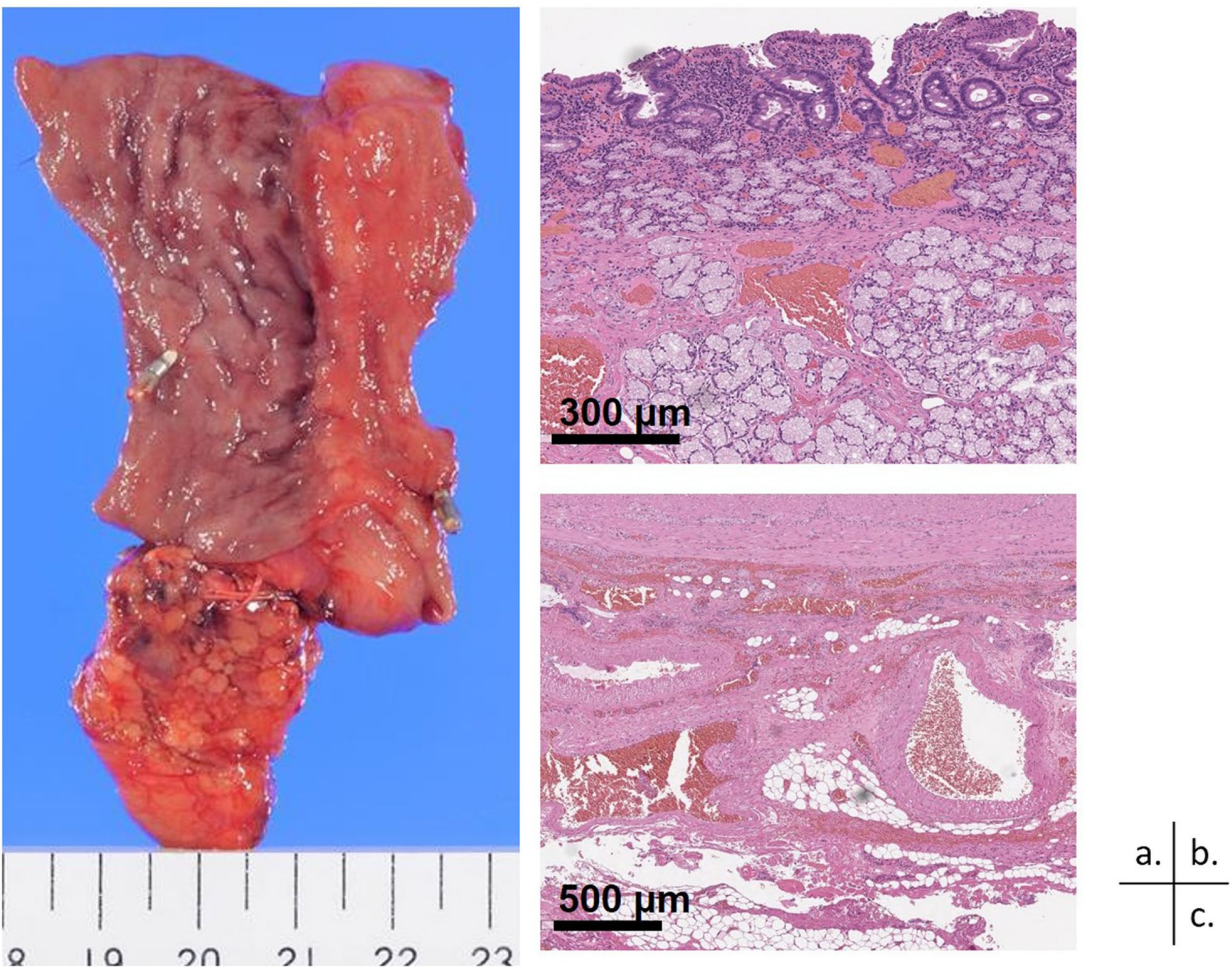
Pathological findings. **a** Macroscopically, about 50 mm of the duodenum is resected. The mucosa of the resected specimen is slightly dark reddish, but is almost intact. **b**, **c** Hematoxylin and eosin staining reveals dilated arteries and veins from the layers of the mucosa to the subserosa

Based on these findings, a subtotal stomach-preserving duodenal bulb resection preceded by IPA and SDA ligation to reduce bleeding during surgery was planned. In addition, intraoperative ICG angiography was planned to determine the final resection line. We prepared to perform pancreas-sparing duodenectomy if the resection line of the duodenum indicated by ICG angiography was anorectal to the duodenal minor papilla and pancreaticoduodenectomy if the resection line was over the duodenal papilla. Exploratory laparotomy via midline incision revealed an expanded right gastric vein, which was presumed to be the effect of the increased blood flow due to AVM. However, no abnormalities were observed in the duodenum. To determine the extent of resection, 12.5 mg of ICG (Daiichi Sankyo Co., Japan) was injected, and ICG imaging was performed using an infrared camera system (SPY-PHI portable handheld imager, Striker, USA). Intraoperative ICG angiography clearly demonstrated the lesion of the AVM, and this area was completely resected after IPA and SDA ligation, as planned preoperatively (Fig. 3). The cut end of the duodenum was sutured and embedded. The anastomosis between the stomach and the jejunum was made by the anterior colonic route. In addition, the Brown anastomosis was added. The operation time was 221 min, and the estimated blood loss was 60 mL. No intraoperative blood transfusion was required. Pathological findings showed dense vascularity from the layers of the mucosa to the subserosa, which was confirmed as an AVM of the duodenum. In addition, extravascular leakage of red blood cells was observed in the subserosa, which was reasonable to consider as a source of GI bleeding (Fig. 4). The postoperative course was uneventful, and he was discharged on postoperative day 11. At the 6-month follow-up, he was doing well and denied any signs of re-bleeding.

## Discussion

AVM is characterized by a nidus, which is an abnormal connection between the arteries and veins without capillaries. No definite pathogenetic explanation has been described for this anomaly. The majority of lesions are congenital and are associated with hereditary hemorrhagic telangiectasia, such as Osler–Weber–Rendu syndrome, whereas an acquired case has also been reported. In the present case, he had no nasal bleeding, vasodilation of body surface, or family history, which are all physical features suggestive of a hereditary disease; thus, we consider him to be a solitary AVM [3, 4]. AVM of the GI tract is clinically important as a cause of bleeding, because angiodysplasias and vascular malformations reportedly cause approximately 5% of non-variceal upper GI tract bleedings [1]. AVM of the GI tract is most often located in the cecum and the right side of the colon; however, AVM located in the duodenum is rare and reported to make up 2.3% of all AVM of the GI tract [5].

The treatment of choice for GI tract AVM is surgical resection of the involved bowel segment with complete resection of the nidus. Because AVMs located in the duodenum or pancreatic head can cause bleeding from the duodenum, there are several case reports in which pancreaticoduodenectomy was performed as treatment [6–9]. To determine the resection area, precise identification of AVM area is important. Intraoperative identification of nidus using ICG angiography in patients with AVM of the central nervous system has been reported in several cases [10]. However, to the best of our knowledge, only three reports of ICG angiography for the GI tract AVM have been reported [11–13], and all of these reports were about AVMs of the small intestine. Therefore, this is the first case of using ICG angiography for duodenal AVM. Since there are important blood vessels and bile ducts running around the duodenum, it is very important to set the exact resection area. In the present case, we successfully performed ICG angiography for intraoperative identification of the duodenal AVM, and we believe that it allowed us to perform precise and organ-preserving surgery in a less invasive manner. On the other hand, ICG angiography prolongs the operation time due to drug administration and observation and may also cause drug allergy. Even with these demerits, we believe that ICG angiography is particularly useful in cases, such as ours, in which a small change in the resection line can significantly affect the surgical procedure.

Another option, treatment with the transarterial embolization (TAE), has been reported as a non-surgical treatment for AVMs [14, 15]. The problem with TAE is that it increases the risk of re-bleeding and portal hypertension due to the development of collateral vessels, and it also includes the risk of enteric necrosis [16]. Furthermore, it is reported that once portal hypertension develops, it is impossible to reduce portal venous hypertension even if the AVM is surgically removed [17]. Considering these factors, we believe that it is effective to stabilize the general condition by TAE for patients with acute gastrointestinal bleeding due to AVM who are in poor general condition, but it is important to perform surgery afterwards. In other reports, TAE was performed preoperatively to reduce blood loss during surgery, not as a curative treatment [18, 19]. However, in our case, we identified the inflow arteries by preoperative angio-CT and successfully controlled intraoperative blood loss by ligating these vessels first. Precise preoperative imaging diagnosis also contributed to the safe performance of surgical operation.

## Conclusions

Intraoperative ICG angiography was useful in identifying the location of the AVM lesion. Using this technique, we could resect the duodenal AVM in the appropriate area.

## Data Availability

The data that support the findings of this study are available from the corresponding author upon reasonable request.

## Abbreviations

AVM: Arteriovenous malformation
GI: Gastrointestinal
ICG: Indocyanine green
CT: Computed tomography
IPA: Inferior pyloric artery
SDA: Superior duodenal artery
TAE: Transarterial embolization
